# Multimodal diagnostic tools and advanced data models for detection of prodromal Parkinson’s disease: a scoping review

**DOI:** 10.1186/s12880-025-01620-5

**Published:** 2025-03-28

**Authors:** Ibrahim Serag, Ahmed Y. Azzam, Amr K. Hassan, Rehab Adel Diab, Mohamed Diab, Mahmoud Tarek Hefnawy, Mohamed Ahmed Ali, Ahmed Negida

**Affiliations:** 1 Faculty of Medicine, Mansoura University, Mansoura, Egypt; 2 Medical Research Group of Egypt, Negida Academy, Arlington, MA USA; 3 Faculty of Medicine, October 6 University, Giza, Egypt; 4 University of California, Irvine, CA USA; 5 Faculty of medicine, Al-Azhar university, Cairo, Egypt; 6 Faculty of Medicine, Alexandria University, Alexandria, Egypt; 7 Faculty of Medicine, Zagazig University, Zagazig, Egypt; 8 Qena Faculty of Medicine, South Valley University, Qena, Egypt; 9 Department of Neurology, Virginia Commonwealth University, Richmond, VA USA

**Keywords:** MRI, ECG, AI, Multimodal diagnostic imaging, Prodromal PD, Scoping review

## Abstract

**Background:**

Parkinson’s Disease (PD) is a progressive neurodegenerative disorder characterized by the loss of dopaminergic neurons in the substantia nigra pars compacta. PD is diagnosed by a combination of motor symptoms including bradykinesia, resting tremors, rigidity and postural instability. Prodromal PD is the stage preceding the onset of classic motor symptoms of PD. The diagnosis of prodromal PD remains challenging despite many available diagnostic modalities.

**Aim:**

This scoping review aims to investigate and explore the current diagnostic modalities used to detect prodromal PD, focusing particularly on multimodal imaging analysis and AI-based approaches.

**Methods:**

We adhered to the PRISMA-SR guidelines for scoping reviews. We conducted a comprehensive literature search at multiple databases such as PubMed, Scopus, Web of Science, and the Cochrane Library from inception to July 2024, using keywords related to prodromal PD and diagnostic modalities. We included studies based on predefined inclusion and exclusion criteria and performed data extraction using a standardized form.

**Results:**

The search included 9 studies involving 567 patients with prodromal PD and 35,643 control. Studies utilized various diagnostic approaches including neuroimaging techniques and AI-driven models. sensitivity ranging from 43 to 84% and specificity up to 96%. Neuroimaging and AI technologies showed promising results in identifying early pathological changes and predicting PD onset. The highest specificity was achieved by neuromelanin-sensitive imaging model, while highest sensitivity was achieved by standard 10-s electrocardiogram (ECG) + Machine learning model.

**Conclusion:**

Advanced diagnostic modalities such as AI-driven models and multimodal neuroimaging revealed promising results in early detection of prodromal PD. However, their clinical application as screening tool for prodromal PD is limited because of the lack of validation. Future research should be directed towards using Multimodal imaging in diagnosing and screening for prodromal PD.

**Clinical trial number:**

Not applicable.

## Introduction

Parkinson’s Disease (PD) is a progressive neurodegenerative disorder characterized by the loss of dopaminergic neurons in the substantia nigra pars compacta [1]. This region of the brain is involved in movement regulation [1]. PD is diagnosed by a combination of motor symptoms including bradykinesia, resting tremors, rigidity and postural instability [2]. Besides these motor symptoms, PD often accompanies non-motor symptoms like sleep disturbance, depression and cognitive impairment [2]. PD is clinically and generally manifest when at least 50% of dopaminergic neurons of substantia nigra are dead and decline in 80% of striatal dopamine levels [3, 4]. PD prevalence increases with age and affecting approximately 10 million patients worldwide and one million patients in America with new incidence estimated by 50,000 new cases annually [5, 6]. There are many risk factors associated with PD such old age, genetic factors, environmental factors, lifestyle, comorbidities like diabetes and biomarkers such as α-synuclein in peripheral tissues and decreased dopamine transporter (DAT) binding [7–9]. There are some theories classifying PD into two subtypes, brain first type in which α-synuclein arise initially in the brain then spreading to peripheral nerves system, the other subtype is body first type in which α-synuclein arise initially in peripheral nerves system or enteric system and spread to brain via gut-brain access [10]. Despite advancements in symptomatic treatment of PD, there is no definitive cure to it as all available treatments only slow its progression [11].

Prodromal Parkinson’s disease is the stage preceding the onset of classic motor symptoms of PD [12]. There are prodromal markers and symptoms that can be associated with prodromal PD such as subtle motor sings, autonomous dysfunction, pathological dopaminergic imaging, depression, REM-sleep behavior disorder (RBD) and non-motor symptoms like hyposmia, constipation and cognitive changes [13]. Additionally, there are certain biomarkers and risk factors are associated with prodromal PD including genetic predisposition, decrease dopamine transporter binding, which can be detected through neuroimaging, and presence of α-synuclein in peripheral tissues [14]. The International Parkinson and Movement Disorder Society (MDS) has been designed and updated the criteria for prodromal PD [7]. However, diagnosis of prodromal PD remains challenging, and the sensitivity and specificity of available diagnostic approaches is still limited [15]. The aim of early detection of prodromal PD is to allow us to include neuroprotective agents and lifestyle modifications that can slow progression of disease and improve long-term outcomes [16]. 

Diagnosis of prodromal PD ranging from traditional clinical assessment to advanced technologies including neuroimaging, biomarker analysis, and artificial intelligence (AI) applications. Neuroimaging modalities such as DAT-SPECT and MRI are used to detection of early changes in brain structures and function [17], some studies investigate the use of biomarkers in blood and cerebrospinal fluid to detect the pathology of prodromal PD [18, 19]. Recently, there are a significant advancement in computational models and AI applications that aim to detection of prodromal PD through machine learning algorithms [20]. These AI models are designed to detect complex patterns across many types of data to improve accuracy of identifying prodromal PD with higher sensitivity and specificity than traditional clinical methods [21]. For example, AI driven analysis of speech patterns can reveal subtle motor abnormalities a long time before clinical diagnosis [22]. AI-driven neuroimaging analysis can effectively detect early dopaminergic dysfunction associated with prodromal PD [23].

Despite advancements of AI models and multimodal imaging analysis in identifying prodromal PD, several gaps in knowledge remain unclear. Most diagnostic modalities have not been fully validated for use in detection and screening for prodromal PD. So, our aim from this scoping review is to systematically investigate and explore the current diagnostic modalities used to detect prodromal PD, with a particular focus on multimodal image analysis and AI-based approaches. We will focus on strengths, limitations and future directions in detection of prodromal PD.

## Methods and materials

### Literature search

We conducted our scoping review adhering to Preferred Reporting Items for Systematic Reviews and Meta-Analyses for Scoping Reviews (PRISMA-SR) checklist [24]. We searched relevant databases including PubMed, Scopus, Web of Science, and the Cochrane Library for eligible studies from inspection to July 2024. We used a combination of keywords and MeSH terms such as “Parkinson’s Disease,” “prodromal Parkinson’s,” “pre-motor symptoms,” “diagnostic modalities,” “early detection.” “Multimodal imaging,” “Artificial intelligence,” “REM sleep behavioral disorder,” “MRI.” “PET scan.” “Early Parkinson*,” “CNN,” and “convolutional neural network,”. This search strategy aimed to identify all relevant studies that investigated diagnostic tools and modalities used in the early detection of prodromal Parkinson’s Disease.

### Inclusion and exclusion criteria

#### Inclusion criteria

1. Articles published in English.
2. Studies focusing on the prodromal stage of Parkinson’s Disease.
3. Research that included validated diagnostic tools (multimodal imaging) or novel AI technologies.
4. Studies report measurable outcomes such as diagnostic accuracy, sensitivity, specificity, and F1 scores.
5. Studies that incorporated a control group for comparison.

#### Exclusion criteria

1. Articles not available in full text.
2. Studies that did not specifically address prodromal Parkinson’s Disease.
3. Literature reviews, case reports, and editorials without original data.

### Data extraction

Data extraction was collected on excel spread sheet using a standardized form designed to capture all relevant data systematically and we performed data extraction by two independent reviewers and any discrepancies between then was resolved by third opinion. This form includes relevant data such as Study ID, study design, country, modality used, sample sizes of prodromal PD patients and controls, age demographics (age at applying modality, age at PD diagnosis and years from applying modality to PD diagnosis), accuracy metrics like sensitivity and specificity, and main findings.

## Results

### Literature search

**Fig. 1:**
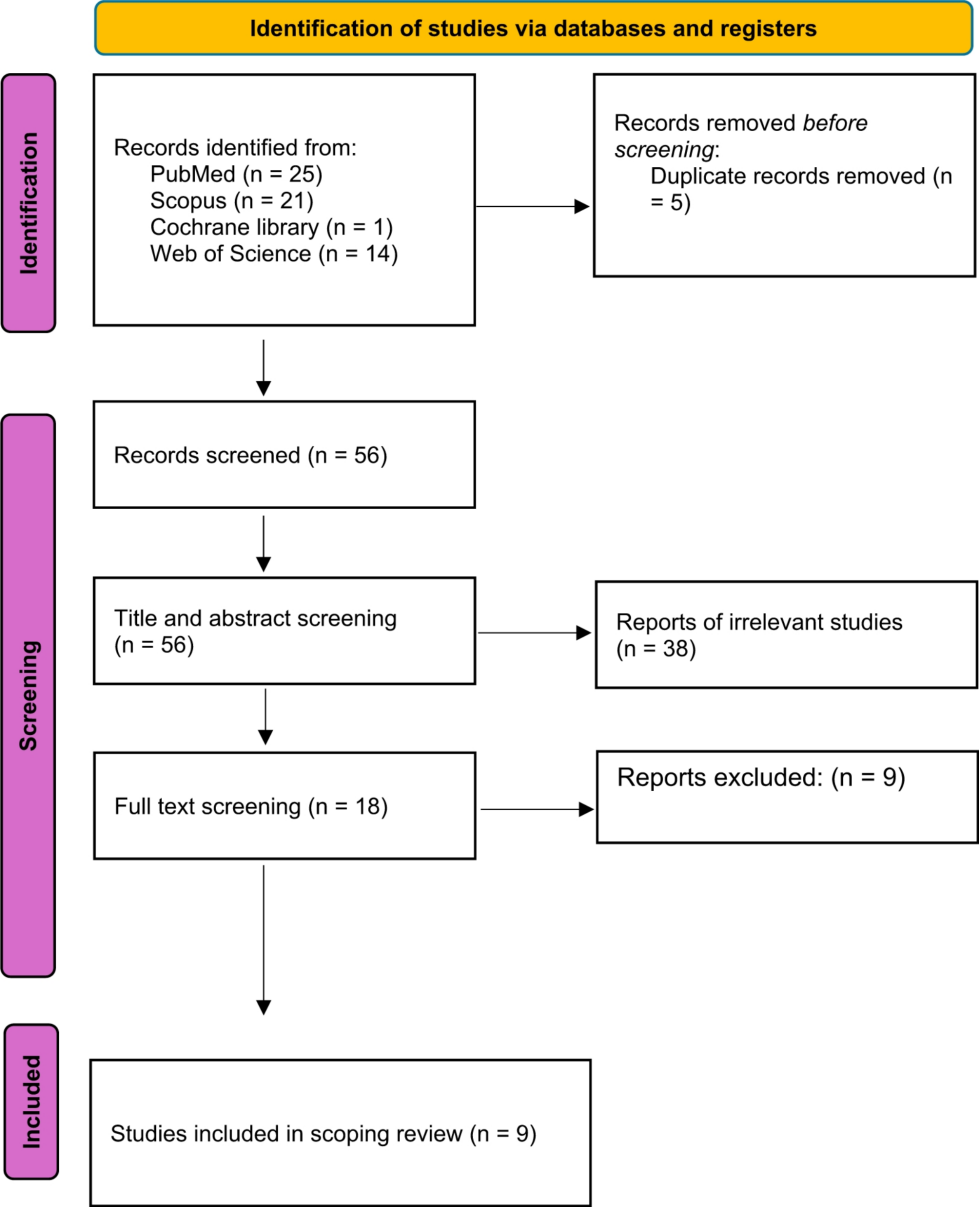
PRISMA flowchart of included studies

Figure 1 shows a flow chart illustrating the process of selecting and including papers according to PRISMA SR guidelines [24]. An electronic search of databases yielded 61 records. Out of these, 56 records were considered for title and abstract screening, while the remaining 5 were found to be duplicates. We performed a title and abstract screening to 56 relevant studies. 38 studies were excluded, and we performed full text screening to 18 studies. After conducting a full text screening, we identified 9 studies that matched our specific criteria for inclusion in our scoping review. These studies involved a total of 567 patients with prodromal PD and 35,643 control (Fig. 1).

### Study characteristics

The systematic search yielded 9 studies meeting our inclusion criteria. These studies originated from various countries including the USA, UK, France, Germany, and China, reflecting a diverse geographical spread. Most of the studies employed cohort designs, with a focus on early diagnostic methods for prodromal Parkinson’s Disease. Sample sizes ranged from 16 to 33,009 participants, and the age of participants ranged from 63 years to 77 years.

### Diagnostic modalities evaluated

We examined different modalities across our included studies that can be classified into:

#### Neuroimaging techniques

five studies utilized advanced imaging techniques such as tensor imaging, neuromelanin-sensitive imaging, electrocardiograms, and multimodal MRI to detect early signs of neurodegeneration.

#### Machine learning models

four studies involve AI-driven models applying these to data from accelerometry, Bayesian network modeling, and voice analysis.

The results of our scoping review highlight the variability in diagnostic performance among different modalities for detecting prodromal PD. Across the included studies, sensitivity ranged from 43 to 84%, with specificity reaching up to 96%. The highest specificity (95%) was achieved using neuromelanin-sensitive imaging, which effectively detected substantia nigra degeneration. The highest sensitivity (84%) was observed in ECG-based AI models, which leveraged machine learning to identify subtle cardiac abnormalities associated with prodromal PD (Table 1).

**Table 1:** Baseline characteristics of included studies

Study ID	Design	Country	Methods used	Sample size		Age at applying method (Mean, SD)		Age at PD diagnosis (Mean, SD)		Years from methods until PD (Mean, SD)	Accuracy	Sensitivity	Specificity	Main findings
				Prodromal PD	Control	Prodromal PD	Control	Prodromal PD	Control	Prodromal PD				
Akbilgic 2020 (28)	Cohort	USA	standard 10-s electrocardiogram (ECG) + Machine learning model	25	25	77.6 (4.9)	78.0 (3.7)	81.9 (4.8)	-	4.3 (2.4)	80%	84%	80%	♦cardiac electrical activity provides important information about the likelihood of future PD not captured by classical HRV metrics.
Schalkamp 2023 (32)	Cohort	UK	Accelerometry + Machine learning models	113	33,009	-	-	-	-	-	NR	- NR	- NR	♦Accelerometry is a potentially important, low-cost screening tool for determining people at risk of developing Parkinson’s disease
Pyatigorskaya 2017 (27)	Cross sectional	France	tensor imaging, neuromelanin (NM)-sensitive imaging and T2* mapping.	19	18	67.3 (8.0)	66.5 (5.1)	-	-	5.7(3.8)	81%	67%	95%	♦NM-sensitive imaging and FA allowed for the detection of SN damage in iRBD patients with good diagnostic accuracy.
Sood 2023 (31)	Cohort	Germany	Bayesian network modeling	18	1178	63 (58, 68)	63 (58, 68)	70 (68, 71)	69 (64, 74)	-	52%	- NR	- NR	♦Accurate PD prediction accounting for the interdependencies in marker profiles could not be derived from the given data. Overall, this work demonstrates the potential of modern AI approaches to advance our understanding of prodromal PD.
Jeancolas 2022 (22)	Cohort	France	supervised learning classifications to automatically detect patients using voice only.	41	98	68.2 (6.3)	60.0 (10.6)	-	-	-	70%	- NR	- NR	♦This study demonstrates the valuable benefit of including automated voice analysis in future diagnostic procedures of prodromal PD.
Ran 2022 (30)	Cohort	China	Brain age vector + Machine learning models	174	171	62.06(7.53)	60.45(11.68)	-	-	-	72.28%	- NR	- NR	♦The proposed brain age vector effectively improves spatial specificity of brain aging measurement and enables individual screening of neurodegenerative diseases.
Peña-Nogales 2019 (26)	Cohort	USA	MRI data (Longitudinal Connectomes)	16	30	67.50(5.19)	65.66 (4.65)	-	-	-	74.40%	68.80%	80%	♦It is possible to quantify neurodegenerative patterns of progression in the prodromal phase with longitudinal diffusion magnetic resonance imaging connectivity data and use these image-based patterns as progression markers for neurodegeneration.
Karabayir 2023 (29)	case–control study	USA	ECG-based fully automatic artifcial intelligence (AI) model to predict PD (1D-CNN model)	131	1058	72.6 (8.0)	69.8 (9.6)	75.3 (7.9)	-	2.7 (1.9)	73%	43%	96%	♦10-s ECGs can effectively classify individuals with prodromal PD in an independent cohort, particularly closer to disease diagnosis. Standard ECGs may help identify individuals with prodromal PD for cost-effective population level early detection and inclusion in disease-modifying therapeutic trials.
Holtbernd 2021 (25)	Cohort	Germany	multimodal MRI.	30	56	66.8 (9.1)	62.9 (11)	-	-	-	- NR	- NR	- NR	♦This study found convergent patterns of structural brain alterations in RBD and PD patients compared to HC. The changes observed suggest a co-occurrence of neurodegeneration and compensatory mechanisms that fail with emerging PD pathology.

Neuroimaging techniques, such as MRI and PET scans, demonstrated potential in detecting early neurodegenerative changes. Diffusion tensor imaging (DTI) and neuromelanin-sensitive MRI were particularly effective in visualizing structural brain alterations, while PET imaging was utilized to assess dopaminergic dysfunction. Machine learning-based approaches applied to accelerometry, voice analysis, and ECG data showed promise in improving diagnostic accuracy by identifying subtle physiological and behavioral markers indicative of prodromal PD.

Among the prodromal PD cohorts included in the studies analyzed, several key syndromes were observed. iRBD was the most studied syndrome, given its strong association with the development of PD. Additionally, hyposmia (olfactory dysfunction) was frequently assessed, as it has been identified as a significant early marker of PD. Other prodromal symptoms, such as autonomic dysfunction and mild motor impairments, were also examined in some studies.

## Discussion

### Advances in multimodal imaging teqniques

#### MRI

In the study conducted by Holtbernd et al., the researchers aimed to characterize structural brain changes in patients with RBD and PD using multimodal MRI. The study included 30 RBD patients, 29 PD patients, and 56 age-matched health controls (HC) who underwent MRI at 3T, encompassing tensor-based morphometry, diffusion tensor imaging, and cortical thickness assessments. The findings revealed that RBD patients exhibited an increased volume in the right caudate nucleus compared to HC and higher cerebellar volume than both PD patients and HC. Additionally, RBD patients showed increased fractional anisotropy (FA) in corticospinal tracts, non-motor related tracts, and cerebellar peduncles compared to both PD patients and HC, while PD patients showed reduced FA in the corpus callosum and lower volumes in the basal ganglia, midbrain, pedunculopontine nuclei, and cerebellum. Conversely, PD patients had increased thalamic volume compared to both RBD patients and HC. These structural alterations suggest a combination of neurodegenerative and compensatory mechanisms, supporting the hypothesis of RBD as a prodromal state of PD. Limitations of the study include gender mismatches among cohorts and the retrospective nature of the study. Despite these limitations, the results enhance the understanding of the continuum between RBD and PD, highlighting the presence of both neurodegeneration and compensatory responses in early PD pathology. Sensitivity and specificity measures were not explicitly stated [25].

In the study conducted by Peña-Nogales et al., the researchers utilized longitudinal diffusion MRI connectome analysis to investigate its potential as a marker for progression in prodromal PD. They employed DTI to create detailed brain connectomes at multiple time points for both prodromal PD patients and healthy controls. The longitudinal connectome progression score, calculated from this diffusion MRI data, provided a measure of changes in brain connectivity over time. The study found that this progression score was significantly higher in prodromal PD patients compared to controls, with an area under the receiver operating characteristic curve (AUC) of 0.89 for distinguishing disease progression and 0.76 for differentiating prodromal PD from controls. These findings suggest that the longitudinal connectome progression score is effective in detecting subtle neurodegenerative changes that precede clinical symptoms of PD. The sensitivity of this approach was reported to be 69%, while the specificity was 80%, indicating that the method is both sensitive to early changes associated with PD and reasonably accurate in identifying healthy individuals. However, this study has several limitations. The data were acquired from a relatively small sample of 51 subjects, with a focus on demographic matching between groups rather than dataset size. This led to the decision to keep 16 prodromal PD subjects as a fully independent set to mitigate overfitting risks. Despite this, the small sample size limits the ability to capture the full heterogeneity of Parkinson’s Disease and the dataset’s applicability to broader populations. Additionally, the use of data from multiple clinical sites introduces potential variability between scanners that could affect the results [26].

A cross-sectional study conducted by Pyatigorskaya et al., aimed to quantify substantia nigra (SN) damage in patients with idiopathic RBD using multimodal MRI techniques, which are crucial for identifying prodromal PD. The research utilized 3-Tesla MRI to perform diffusion tensor imaging, neuromelanin (NM)-sensitive imaging, and T2* mapping, analyzing SN volumes and signal intensities. The study included 19 iRBD patients and 18 controls, and the results revealed significant reductions in NM-sensitive SN volume and signal intensity, alongside a decrease in fractional anisotropy (FA) in iRBD patients compared to controls. These biomarkers demonstrated strong diagnostic accuracy, with NM-sensitive imaging and FA showing accuracies of 0.86 and 0.77 respectively, and a combined accuracy of 0.92. Visual assessment by two raters also exhibited substantial agreement, with a diagnostic accuracy of 0.81. However, the study has notable limitations. The cross-sectional design restricts the ability to predict future development of PD, and the relatively small sample size of 19 iRBD patients necessitates validation in larger cohorts. Additionally, the study did not include DAT-SPECT imaging, which is important for correlating NM loss with striatal dopaminergic denervation [27].

#### Electrocardiogram (ECG)

The study conducted by Akbilgic et al. presents an innovative approach for identifying individuals at high risk of developing PD through ECG analysis. The research focuses on the application of Probabilistic Symbolic Pattern Recognition (PSPR) to ECG data, alongside traditional heart rate variability (HRV) metrics, to uncover early indicators of PD. The study involved 60 Japanese American males, including 10 with prevalent PD, 25 with prodromal PD, and 25 controls, ensuring a well-defined comparative group. The researchers employed standard 10-second ECG recordings and extracted various features using both classical and advanced methods. PSPR involves discretizing ECG signals into symbols, then comparing these symbolic patterns using probabilistic models to detect deviations indicative of prodromal PD. The application of PSPR yielded significant results, with four PSPR-derived features selected through stepwise logistic regression as predictors of PD. The final regression model demonstrated an impressive AUC of 0.90, with a five-fold cross-validation producing an average AUC of 0.835. This suggests that PSPR is particularly effective in capturing subtle changes in cardiac electrical activity preceding the motor symptoms of PD, which traditional HRV metrics failed to detect. Despite its promising results, the study acknowledges limitations such as the small sample size and the exclusion of participants with major cardiovascular conditions or those on specific medications. These factors may influence the generalizability of the findings, which were confined to a demographic of Japanese American men [28].

A case control study conducted by Karabayir et al., aimed to develop a deep learning model for identifying prodromal PD using ECG data, leveraging a one-dimensional convolutional neural network (1D-CNN) to predict PD risk up to five years before diagnosis. The research, which used ECGs from Loyola University Chicago (LUC) and University of Tennessee-Methodist Le Bonheur Healthcare (MLH), involved training the 1D-CNN model on 90% of the MLH dataset (131 PD cases and 1058 controls) and validating it internally before external validation on the LUC dataset (29 PD cases and 165 controls). The 1D-CNN model’s performance varied with the time interval from the ECG recording to PD diagnosis. It achieved an area under the curve (AUC) of 0.74 for predicting PD within 6 months to 1 year, 0.69 for 6 months to 3 years, and 0.67 for 6 months to 5 years. The model demonstrated moderate sensitivity and high specificity, with sensitivity ranging from 50 to 70% depending on the prediction time frame, and specificity consistently above 90%. The 1D-CNN model outperformed traditional models based on feature engineering, which had lower AUC values, indicating its superior capability in early PD detection. Subgroup analyses revealed variations in prediction accuracy based on sex, race, and age, with limitations including the potential misclassification of PD cases and the use of different ECG data systems. The study underscores the promise of deep learning models in enhancing early PD detection and highlights the need for further refinement and validation using larger, well-annotated datasets [29].

### Advances in artificial intelligence teqniques

Ran et al. presented a novel approach for measuring brain aging through the development of a “brain age vector,” which enhances spatial specificity in the context of neurodegenerative disorders. Traditional methods of brain age estimation, which often rely on a single brain age gap, may overlook regional variations in brain aging that are crucial for early disease detection. By integrating brain age modeling with Shapley Additive Explanations (SHAP), they created a brain age vector that attributes regional contributions to overall brain age, thereby capturing spatial patterns of pathological aging. Their methodology involved training a brain age model using volumetric brain features extracted from multiple public datasets, followed by SHAP analysis to generate the brain age vector for each subject. This approach was evaluated on groups of normal aging, prodromal PD, stable mild cognitive impairment (sMCI), and progressive mild cognitive impairment (pMCI), and compared against other brain aging metrics such as the single brain age gap and regional brain age gaps. The brain age vector demonstrated significant improvements in detecting disorder-specific aging patterns, with notable regional abnormalities observed in the medial temporal lobe and striatum for prodromal Alzheimer’s disease (AD) and PD, respectively. The brain age vector’s efficacy in early disease screening was further confirmed through high AUC values of 83.39% for pMCI and 72.28% for prodromal PD, highlighting its potential as a robust tool for neurodegenerative disease identification. Additionally, the brain age vector exhibited high test-retest reliability and was consistent with XGBoost’s feature importance results, underscoring its reliability and the accuracy of feature attribution. This novel approach provides enhanced spatial specificity in brain aging measurement and offers valuable insights for neurodegenerative disease screening and monitoring [30].

In Jeancolas et al. study, they explored voice characteristics as potential biomarkers for detecting early PD from the prodromal stage of iRBD through automated acoustic analysis. The researchers analyzed voice samples from 256 French speakers, including 117 with early PD, 41 with iRBD, and 98 healthy controls. Utilizing a variety of recording devices and speech tasks, they extracted high-level features related to prosody, phonation, speech fluency, and rhythm. The analysis revealed significant PD-related impairments in prosody, pause durations, and rhythmic abilities, with more pronounced changes in males compared to females. Early PD detection achieved balanced accuracies of 89% in males and 70% in females, while iRBD detection accuracy reached 63%, improving to 70% among iRBD patients with mild motor symptoms. The study highlights that automated voice analysis could be a valuable tool in diagnosing prodromal PD and emphasizes the importance of considering sex differences in voice-related impairments. The findings suggest that while voice characteristics are promising for detecting PD, the accuracy varies between genders and stages of the disease, and the inclusion of automated voice analysis could enhance early diagnostic procedures for Parkinson’s disease. And one of this study’s limitation is that groups of study were not totally age matched [22].

In the study by Sood et al., Bayesian network (BN) modeling was employed to elucidate the interdependencies among risk and prodromal markers of PD using data from TREND study. This study involved 1178 healthy, PD-free individuals and 24 incident PD cases, with data collected over up to 10 years. The BN approach addressed limitations of traditional PD prediction models by modeling the probabilistic dependencies between 18 markers, including autonomic dysfunction, lifestyle factors, environmental exposures, neuropsychiatric features, and neurological markers. The BN revealed robust interdependencies, such as age’s link to subthreshold parkinsonism and urinary dysfunction, sex’s association with SN hyper echogenicity and depression, and depression’s connection to symptomatic hypotension and excessive daytime somnolence. Novel associations were also identified, including non-smoking’s link to depression and solvent exposure’s connection to symptomatic hypotension. Evaluation of the BN involved generating synthetic data and assessing its realism through a random forest classifier, achieving a partial AUC of 52%, indicating marginal improvement over chance. Predictive modeling showed a ~ 10% reduction in AUC when using synthetic data compared to real data, reflecting some loss in prediction accuracy. Overall, this methodology provided a more comprehensive understanding of how various markers interrelate in the prodromal phase of PD [31].

The study by Schalkamp et al. explored the potential of wearable accelerometry data to identify prodromal PD years before clinical diagnosis. Using data from the UK Biobank, the researchers developed machine learning models trained on accelerometric data, which were compared against models using genetic, lifestyle, blood biochemistry, and symptom data. The accelerometric models demonstrated superior performance, with higher accuracy, sensitivity, and specificity in distinguishing PD cases and prodromal PD from the general population. Specifically, the models showed a significant reduction in movement associated with PD, detectable several years before clinical diagnosis, and accurately predicted both the presence of PD and the timing of diagnosis. The study found that PD patients exhibited poorer sleep quality compared to other diagnostic groups. However, the study’s limitations include the lack of external replication, which may affect the generalizability of the findings. Despite this, the results highlight accelerometry as a promising tool for the early detection and monitoring of prodromal PD [32].

Zhang et al. (2024) highlights the significance of considering early-stage PD beyond traditional prodromal markers, specifically through the PD with normal cognition (PD-NC) classification. Their study demonstrated distinct morphological changes in the hippocampus and amygdala in PD-NC patients, suggesting that neurodegenerative alterations begin even before significant cognitive decline occurs. These findings emphasize the need for a broader approach in defining prodromal PD, incorporating structural brain changes beyond standard motor and non-motor symptoms [33].

### Future directions for research

As we look to the future of detecting prodromal PD, the integration of multimodal imaging, ECG analysis, and AI offers promising avenues for advancement. Despite significant progress, there remains a pressing need to refine diagnostic tools and improve early detection strategies. Multimodal imaging, which combines various imaging techniques such as MRI, PET, and DTI, provides a comprehensive view of neurodegenerative changes that precede the onset of clinical symptoms. For instance, combining neuromelanin-sensitive MRI with diffusion tensor imaging can enhance the detection of subtle brain alterations associated with prodromal PD. Additionally, ECG-based AI models have demonstrated potential in detecting prodromal PD through subtle cardiac abnormalities, providing a non-invasive and cost-effective screening method. Future technological pathways should focus on developing standardized protocols for integrating multimodal imaging with AI-driven analytics. Large-scale validation studies are essential to ensure that AI algorithms trained on different datasets generalize well across diverse populations. The expansion of deep learning techniques, particularly explainable AI models, could improve the interpretability of results and foster trust in AI-assisted diagnostics.

Despite the promising advancements of AI-based prediction models for prodromal PD, several limitations must be addressed before these techniques can be effectively integrated into clinical practice. One major challenge is data heterogeneity, as AI models are often trained on datasets with limited demographic diversity, making generalizability a concern. Future studies should prioritize the development of standardized, multi-center datasets to improve model robustness across different populations.

## Conclusion

This scoping review underscores the potential of advanced diagnostic modalities, particularly multimodal imaging and AI-based approaches, in enhancing the early detection of prodromal Parkinson’s Disease. While these technologies demonstrate promising results, their clinical implementation is hindered by limitations in validation and standardization. Future research should prioritize the integration of multimodal imaging techniques with AI models to improve diagnostic accuracy and early detection. Large-scale, longitudinal studies are essential to validate these tools and refine their application in clinical settings. By addressing these challenges, we can advance the early identification of prodromal PD, ultimately leading to better management and improved patient outcomes.

## Data Availability

All data generated or analyzed during this study are included in this published article.

## References

[1] Ramesh S, Arachchige ASPM. Depletion of dopamine in Parkinson’s disease and relevant therapeutic options: A review of the literature. AIMS Neurosci. 2023;10(3):200–31. 37841347 10.3934/Neuroscience.2023017 PMC10567584

[2] Kouli A, Torsney KM, Kuan W-L. Parkinson’s disease: etiology, neuropathology, and pathogenesis. In: Stoker TB, Greenland JC, editors. Parkinson’s disease: pathogenesis and clinical aspects. Brisbane (AU): Codon. 2018. 30702842

[3] Ross GW, Abbott RD, Petrovitch H, Tanner CM, White LR. Pre-motor features of Parkinson’s disease: the Honolulu-Asia aging study experience. Parkinsonism Relat Disord. 2012;18(Suppl 1):S199–202. 22166434 10.1016/S1353-8020(11)70062-1

[4] Heng N, Malek N, Lawton MA, Nodehi A, Pitz V, Grosset KA, et al. Striatal dopamine loss in early Parkinson’s disease: systematic review and novel analysis of dopamine transporter imaging. Mov Disord Clin Pract (Hoboken). 2023;10(4):539–46. 37070042 10.1002/mdc3.13687 PMC10105104

[5] Goldman SM. Environmental toxins and Parkinson’s disease. Annu Rev Pharmacol Toxicol. 2014;54:141–64. 24050700 10.1146/annurev-pharmtox-011613-135937

[6] Willis AW, Roberts E, Beck JC, Fiske B, Ross W, Savica R, et al. Incidence of Parkinson disease in North America. Npj Parkinsons Disease. 2022;8(1):170. 10.1038/s41531-022-00410-y PMC9755252 36522332

[7] Berg D, Postuma RB, Adler CH, Bloem BR, Chan P, Dubois B, et al. MDS research criteria for prodromal Parkinson’s disease. Mov Disord. 2015;30(12):1600–11. 26474317 10.1002/mds.26431

[8] Prajjwal P, Flores Sanga HS, Acharya K, Tango T, John J, Rodriguez RSC, et al. Parkinson’s disease updates: addressing the pathophysiology, risk factors, genetics, diagnosis, along with the medical and surgical treatment. Ann Med Surg (Lond). 2023;85(10):4887–902. 37811009 10.1097/MS9.0000000000001142 PMC10553032

[9] Chahine LM, Beach TG, Adler CH, Hepker M, Kanthasamy A, Appel S, et al. Central and peripheral α-synuclein in Parkinson disease detected by seed amplification assay. Ann Clin Transl Neurol. 2023;10(5):696–705. 36972727 10.1002/acn3.51753 PMC10187727

[10] Xu Z, Hu T, Xu C, Liang X, Li S, Sun Y, et al. Disease progression in proposed brain-first and body-first Parkinson’s disease subtypes. Npj Parkinsons Disease. 2024;10(1):111. 10.1038/s41531-024-00730-1 PMC11150376 38834646

[11] Parkinson’s Disease. Challenges, Progress, and Promise| National Institute of Neurological Disorders and Stroke. [cited 2024 Jul 27]. Available from: https://www.ninds.nih.gov/current-research/focus-disorders/parkinsons-disease-research/parkinsons-disease-challenges-progress-and-promise

[12] Heinzel S, Roeben B, Ben-Shlomo Y, Lerche S, Alves G, Barone P, et al. Prodromal markers in Parkinson’s disease: limitations in longitudinal studies and lessons learned. Front Aging Neurosci. 2016;8:147. 27445791 10.3389/fnagi.2016.00147 PMC4916171

[13] Hustad E, Aasly JO. Clinical and imaging markers of prodromal Parkinson’s disease. Front Neurol. 2020;11:395. 32457695 10.3389/fneur.2020.00395 PMC7225301

[14] Du T, Wang L, Liu W, Zhu G, Chen Y, Zhang J. Biomarkers and the role of α-Synuclein in Parkinson’s disease. Front Aging Neurosci. 2021;13:645996. 33833675 10.3389/fnagi.2021.645996 PMC8021696

[15] Jackson H, Anzures-Cabrera J, Simuni T, Postuma RB, Marek K, Pagano G. Identifying prodromal symptoms at high specificity for Parkinson’s disease. Front Aging Neurosci. 2023;15:1232387. 37810617 10.3389/fnagi.2023.1232387 PMC10556459

[16] Mahlknecht P, Marini K, Werkmann M, Poewe W, Seppi K. Prodromal Parkinson’s disease: hype or hope for disease-modification trials? Transl Neurodegener. 2022;11(1):11. 35184752 10.1186/s40035-022-00286-1 PMC8859908

[17] Bidesi NSR, Vang Andersen I, Windhorst AD, Shalgunov V, Herth MM. The role of neuroimaging in Parkinson’s disease. J Neurochem. 2021;159(4):660–89. 34532856 10.1111/jnc.15516 PMC9291628

[18] Zhang Q, Wang H, Shi Y, Li W. White matter biomarker for predicting de Novo Parkinson’s disease using tract-based Spatial statistics: a machine learning-based model. Quant Imaging Med Surg. 2024;14(4):3086–106. 38617147 10.21037/qims-23-1478 PMC11007501

[19] Tönges L, Buhmann C, Klebe S, Klucken J, Kwon EH, Müller T, et al. Blood-based biomarker in Parkinson’s disease: potential for future applications in clinical research and practice. J Neural Transm. 2022;129(9):1201–17. 35428925 10.1007/s00702-022-02498-1 PMC9463345

[20] Lai H, Li X-Y, Xu F, Zhu J, Li X, Song Y et al. Applications of machine learning to diagnosis of Parkinson’s disease. Brain Sci. 2023;13(11). 10.3390/brainsci13111546 PMC10670005 38002506

[21] Dixit S, Bohre K, Singh Y, Himeur Y, Mansoor W, Atalla S, et al. A comprehensive review on AI-Enabled models for Parkinson’s disease diagnosis. Electronics. 2023;12(4):783.

[22] Jeancolas L, Mangone G, Petrovska-Delacrétaz D, Benali H, Benkelfat B-E, Arnulf I, et al. Voice characteristics from isolated rapid eye movement sleep behavior disorder to early Parkinson’s disease. Parkinsonism Relat Disord. 2022;95:86–91. 35063866 10.1016/j.parkreldis.2022.01.003

[23] Zarkali A, Thomas GEC, Zetterberg H, Weil RS. Neuroimaging and fluid biomarkers in Parkinson’s disease in an era of targeted interventions. Nat Commun. 2024;15(1):5661. 38969680 10.1038/s41467-024-49949-9 PMC11226684

[24] Tricco AC, Lillie E, Zarin W, O’Brien KK, Colquhoun H, Levac D, et al. PRISMA extension for scoping reviews (PRISMA-ScR): checklist and explanation. Ann Intern Med. 2018;169(7):467–73. 30178033 10.7326/M18-0850

[25] Holtbernd F, Romanzetti S, Oertel WH, Knake S, Sittig E, Heidbreder A et al. Convergent patterns of structural brain changes in rapid eye movement sleep behavior disorder and Parkinson’s disease on behalf of the German rapid eye movement sleep behavior disorder study group. Sleep. 2021;44(3). 10.1093/sleep/zsaa199 32974664

[26] Peña-Nogales Ó, Ellmore TM, de Luis-García R, Suescun J, Schiess MC, Giancardo L. Longitudinal connectomes as a candidate progression marker for prodromal Parkinson’s disease. Front Neurosci. 2018;12:967. 30686966 10.3389/fnins.2018.00967 PMC6333847

[27] Pyatigorskaya N, Gaurav R, Arnaldi D, Leu-Semenescu S, Yahia-Cherif L, Valabregue R et al. Magnetic resonance imaging biomarkers to assess substantia Nigra damage in idiopathic rapid eye movement sleep behavior disorder. Sleep. 2017;40(11). 10.1093/sleep/zsx149 28958075

[28] Akbilgic O, Kamaleswaran R, Mohammed A, Ross GW, Masaki K, Petrovitch H, et al. Electrocardiographic changes predate Parkinson’s disease onset. Sci Rep. 2020;10(1):11319. 32647196 10.1038/s41598-020-68241-6 PMC7347531

[29] Karabayir I, Gunturkun F, Butler L, Goldman SM, Kamaleswaran R, Davis RL, et al. Externally validated deep learning model to identify prodromal Parkinson’s disease from electrocardiogram. Sci Rep. 2023;13(1):12290. 37516770 10.1038/s41598-023-38782-7 PMC10387090

[30] Ran C, Yang Y, Ye C, Lv H, Ma T. Brain age vector: A measure of brain aging with enhanced neurodegenerative disorder specificity. Hum Brain Mapp. 2022;43(16):5017–31. 36094058 10.1002/hbm.26066 PMC9582375

[31] Sood M, Suenkel U, von Thaler A-K, Zacharias HU, Brockmann K, Eschweiler GW, et al. Bayesian network modeling of risk and prodromal markers of Parkinson’s disease. PLoS ONE. 2023;18(2):e0280609. 36827273 10.1371/journal.pone.0280609 PMC9955606

[32] Schalkamp A-K, Peall KJ, Harrison NA, Sandor C. Wearable movement-tracking data identify Parkinson’s disease years before clinical diagnosis. Nat Med. 2023;29(8):2048–56. 37400639 10.1038/s41591-023-02440-2

[33] Zhang L, Zhang P, Dong Q, Zhao Z, Zheng W, Zhang J, et al. Fine-grained features characterize hippocampal and amygdaloid change pattern in Parkinson’s disease and discriminate cognitive-deficit subtype. CNS Neurosci Ther. 2024;30(1):e14480. 37849445 10.1111/cns.14480 PMC10805398

